# On the Extent and Origins of Genic Novelty in the Phylum Nematoda

**DOI:** 10.1371/journal.pntd.0000258

**Published:** 2008-07-02

**Authors:** James Wasmuth, Ralf Schmid, Ann Hedley, Mark Blaxter

**Affiliations:** 1 Institute of Evolutionary Biology, University of Edinburgh, Edinburgh, United Kingdom; 2 Program for Molecular Structure and Function, Hospital for Sick Children, Toronto, Ontario, Canada; 3 Department of Biochemistry, University of Leicester, Leicester, United Kingdom; University of Pittsburgh, United States of America

## Abstract

**Background:**

The phylum Nematoda is biologically diverse, including parasites of plants and animals as well as free-living taxa. Underpinning this diversity will be commensurate diversity in expressed genes, including gene sets associated specifically with evolution of parasitism.

**Methods and Findings:**

Here we have analyzed the extensive expressed sequence tag data (available for 37 nematode species, most of which are parasites) and define over 120,000 distinct putative genes from which we have derived robust protein translations. Combined with the complete proteomes of *Caenorhabditis elegans* and *Caenorhabditis briggsae*, these proteins have been grouped into 65,000 protein families that in turn contain 40,000 distinct protein domains. We have mapped the occurrence of domains and families across the Nematoda and compared the nematode data to that available for other phyla. Gene loss is common, and in particular we identify nearly 5,000 genes that may have been lost from the lineage leading to the model nematode *C. elegans*. We find a preponderance of novelty, including 56,000 nematode-restricted protein families and 26,000 nematode-restricted domains. Mapping of the latest time-of-origin of these new families and domains across the nematode phylogeny revealed ongoing evolution of novelty. A number of genes from parasitic species had signatures of horizontal transfer from their host organisms, and parasitic species had a greater proportion of novel, secreted proteins than did free-living ones.

**Conclusions:**

These classes of genes may underpin parasitic phenotypes, and thus may be targets for development of effective control measures.

## Author Summary

The high-throughput sequencing of messenger RNA from parasitic organisms has permitted large-scale sequence analyses typically reserved for complete genome studies. Such expressed sequence tags (ESTs) have previously been generated for 37 species from the phylum Nematoda, of which 35 were from parasitic species. These datasets were combined with the complete genomes of *Caenorhabditis elegans* and *C. briggsae*. The sequences were assembled into 65,000 protein families, and decorated with 40,000 distinct protein domains. These annotations were analysed in the context of the nematode phylogeny. We identified massive gene loss in the model nematode, *C. elegans*, as well as plant-like proteins in nematodes that cause crop damage. Furthermore, many protein families were found in small groups of closely related species and may represent innovations necessary to sustain their parasitic ecologies. All of these data are presented at NemBase (www.nematodes.org) and will aid researchers working on this important group of parasites.

## Introduction

The vast majority of species are unlikely to be selected for whole genome sequencing, whatever their importance in terms of evolution, health and ecology. The few eukaryote species selected for such projects, despite their utility in laboratory investigation, are unlikely to be representative of the genomic diversity of speciose phyla. For example, Arthropoda and Nematoda have over one million species each [1],[2] and the ∼20 genomes completed [3]–[7] or in sequencing will illuminate only small parts of their diversity. Expressed sequence tags (ESTs) have proved to be a cost-effective and rapid method for identification of the genes from a target species [8]. Although the largest EST collections have been generated primarily for the annotation of complete genome sequences (e.g. human and mouse), more than half the sequences in GenBank's EST depository (dbEST) [9] are from otherwise neglected genomes. One phylum that has benefited from an EST sequencing approach is the Nematoda [10]–[13].

Nematodes (or round worms) are abundant and diverse in terms of biology and ecology [14]. They are ubiquitous members of the meiofauna and play a core role in nutrient recycling. Parasitic species of this phylum are the causative agents of six of the thirteen neglected tropical diseases which afflict around 2.7 billion people [15]–[19]. The diseases caused by nematodes are extremely varied, and include anaemia and malnutrition (caused by hookworms such *Ancylostoma ceylanicum*), African river blindness (caused by the filarial nematode *Onchocerca volvulus*) and elephantiasis (caused by the filarial nematode *Brugia malayi*). In terms of disability adjusted life years (DALYS), the burden of lymphatic filariasis (5.8 million DALYs), onchocerciasis (0.5 million DALYs) and intestinal nematode infections (3 million DALYs) is significant. Among school aged children (5–14), the impact of intestinal nematodes is even greater than malaria [20]. Parasites are also responsible for substantial losses in agriculture. Plant-parasitic nematodes, such as the root-knot nematodes (*Meloidogyne* spp.), are major crop pathogens throughout the world, impacting both the quantity and quality of marketable yields, causing an estimated US$80bn in damage annually [21], and parasites of livestock are the cause for severe economic losses. The fully sequenced genomes of the free-living nematodes *C. elegans* and *C. briggsae* makes the analysis of EST datasets from parasitic nematode species particularly informative, in that both elements of core biology and particular adaptations specific to parasitism can be investigated.

Already more than a dozen species- or family-specific analyses of nematode EST datasets have been published, considering parasites of humans [22]–[24], animals [25]–[27] and plants [28],[29]. The first whole-phylum meta-analysis was based on 265,000 sequences from 30 species, defining 93,645 putative genes [11]. Surprisingly, 30–70% of each species' dataset was found to have no significant similarity (as defined by BLAST searches) with any other sequence either within or outwith the sampled nematodes. Do these sequences define new genes, with new functions in nematodes? Or are they transcriptional noise derived from non-coding sequence with no functional significance? The majority of functional annotations have been assigned through sequence similarity to other proteins [30], and thus a large number of nematode proteins lack clues as to their importance to the organism's survival. In the absence of annotation, these data are limited in their practical use, for example, in identifying the lead novel targets for anthelminthic drugs.

One indication of a gene's significance, in worm survival, is its presence in a number of nematode species. Proteins with essential, conserved functions will tend to be conserved between species, and thus will be members of protein families. Protein families restricted to the Nematoda, but found in a number of species, invite further study to reveal their function. Proteins often share local regions or similarity despite being non-orthologous [31], with the interplay between these domains underpinning their function. There are a number of widely used protein domain databases [32]–[35] which provide domain models to search. In addition, it is possible to identify new domains through similarity searches [36], and nematode-restricted novel domains may yield novel insights into avenues for control of parasites.

EST datasets have been considered less than ideal for such analyses, due to the occurrence of frame-shifts, ambiguous base calls and untranslated regions [37]–[39]. However, coding regions can be accurately predicted from EST cluster consensuses using a hierarchical approach such as that employed by prot4EST [39]. A great deal of care must be taken when translating sequences that do not have sequence similarity to known proteins. ESTScan, incorporated in the prot4EST pipeline, locates (and corrects) coding regions through the identification of frames that have oligonucleotide frequencies resembling those of the training dataset. However, by definition few sequence data are available in the public repositories for neglected species such as parasitic nematodes.

Here we have inferred protein translations for over 120,000 putative genes from EST data from 37 species of nematodes using both high quality codon usage tables for each species [40] and synthetic training sets. This protein dataset, NemPep3, is employed here to investigate protein family (NemFam3) and protein domain (NemDom3) composition of nematodes, and presented in an online database NEMBASE3. Our key findings are:

- the definition of protein domains apparently unique to Nematoda;
- the mapping of the latest time-of-origin of these new families and domains across the nematode phylogeny, revealing ongoing ‘invention’ of novelty;
- the discovery in parasitic species of genes with signatures of horizontal transfer from their host organisms;
- the demonstration of gene loss, particularly of many genes lost from the lineage leading to the model nematode *C. elegans*.

## Materials and Methods

### Generating NEMBASE3 and NemPep3

Sequence data were sourced from EMBL/GenBank/DDBJ and from WormBase (http://www.wormbase.org) as follows:

#### Nematode ESTs

**Figure 1:**
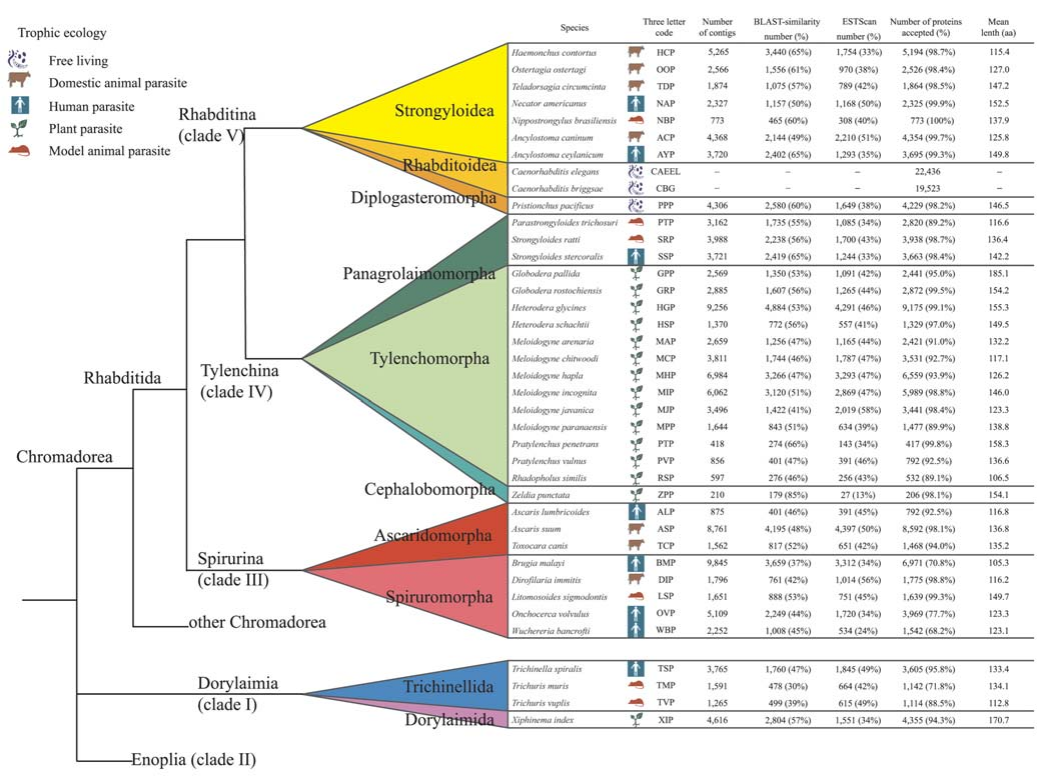
Nematode species contributing to NemPep3. EST cluster consensuses (putative genes) from 37 nematode species were obtained from NEMBASE3. This set of species includes seven not previously analyzed [11]. The species are organized by their systematic grouping based on the SSU rRNA phylogeny [14]. Feeding strategy is indicated by the small icons. We use contig to describe the consensus sequence produced for each set of clustered ESTs. For each species, the numbers of peptides derived from the BLAST-similarity and ESTScan methods of prot4EST [39] are given: only polypeptides generated by these two high-quality components contributed to NemPep3. The complete proteomes of *C. elegans* and *C. briggsae* were obtained from WormBase.

Nematode ESTs, produced by a number of projects including the Washington University Nematode Genomics Programme and the Edinburgh-Sanger Institute Nematode EST Program, were downloaded from EMBL/GenBank/DDBJ (May 2005) and processed to generate the core data for NEMBASE release 3 (NEMBASE3) using the PartiGene suite of programs [39],[41]. Briefly, PartiGene filters sequences for vector and other contaminants, clusters them into putative gene objects using CLOBB [42], and predicts consensus sequences using phrap [43],[44]. The clustering in NEMBASE3 is an incremental update of clusters previously reported in NEMBASE2 [11],[12]. Complete proteomes for *C. elegans* and *C. briggsae* were derived from WormBase (http://www.wormbase.org/). The nematode species analyzed, and the three-letter codes used to designate clusters are given in Figure 1.

#### Peptide prediction

NemPep (version 3) was built from NEMBASE3 using prot4EST (version 2.2) [39]. prot4EST uses three databases (ribosomal RNAs (rRNAs), mitochondrial genomes, and a comprehensive protein database) and custom codon usage tables to filter and translate EST consensus sequences. The sequences for the rRNA database were obtained from the European rRNA database [45]. The E-value cut off for the BLASTN search was 1e-65. For the mitochondrial database, all available proteins of mitochondrial genomes from metazoan lineages were extracted from GenBank using a script written by Martin Jones. This set of sequences was reduced in complexity so that no two sequences shared more than 70% identity. The E-value cut off for the mitochondrial BLASTX search was 1e-8. The protein database used was UniRef100 (version 4) available through UniProt knowledgebase [46]. UniRef BLASTX searches [47] used an E-value cut off of 1e-8.

#### Codon usage tables

The Codon Usage Database offers tables for most of the species studied here [48]. However, none of them could be considered representative as they are built from a small number of codons. It was important to sure accurate codon usage for each species, as our recent study identified extreme differences in base composition among species: e.g. *S. ratti* has ∼10% GC at the third position while *R. similis* has ∼64% GC [40]. We built more comprehensive tables, using conserved segments identified from BLAST comparisons to the UniProt database. The matched regions (E-value cut off 1e-8) were extracted and processed using custom Perl scripts, making use of the *cusp* program from EMBOSS [49].

#### ESTScan Matrices

The codon usage tables, described above were used to generate synthetic training sets for ESTScan [38]. Wormpep (version 140) was used as the template proteome, which was reverse-translated with a Perl script.

#### NemPep3

All EST clusters were analyzed using prot4EST, but only those yielding translations with the BLAST-based or ESTScan methods were incorporated into NemPep3, as translations using ‘longest open reading frame’ were of generally lower quality. NemPep3 entries are designated by three letter codes ending with the letter ‘P’ to signify that these are peptide objects, distinguishing them from EST cluster objects (‘C’).

### Defining protein families from the NemPep3 database: Production of NemFam3

We used TRIBE-MCL to generate protein families from NemPep3 [50]. In TRIBE-MCL, the Inflation parameter defines the tightness of the clusters. No single Inflation parameter value will correctly return all protein families, just as no single molecular clock exists to describe the evolution of all genes. Therefore we repeated the clustering procedure over a range of values and recorded all the clusters, following a previous study of prokaryote proteins [51]. The input to TRIBE-MCL was an all-against-all BLAST report. The number of families generated varied from 42,865 to 71,867. All five sets of protein families are stored in NemBase3.

We used NemFam3 to investigate how sampling from additional species affected the discovery of protein families, generating a “collector's curve” of discovery of novelty. First we took those families for which the only nematode species present was *C. elegans*. We then added new families identified in each species in turn, adding them in the approximate order of their phylogenetic distance from *C. elegans*: Rhabditoidea (CBG; see Figure 1 for three letter species codes); Strongyloidea (ACP, AYP, HCP, NAP, NBP, OOP, TDP); Diplogasteromorpha (PPP); Panagrolaimomorpha (PTP, SRP, SSP); Tylenchomorpha (GPP, GRP, HGP, HSP, MAP, MCP, MHP, MIP, MPP, PEP, PVP, RSP); Cephalobomorpha (ZPP); Ascaridomorpha (ALP, ASP, TCP); Spiruromorpha (BMP, DIP, LSP, OVP, WBP); Trichinellida (TMP, TVP, TSP); Dorylaimida (XIP).

### Biochemical pathway analysis of nematode proteomes

All EST derived proteins were annotated with matches to the KEGG database [52] with a script developed in house which makes use of BLAST comparisons. We wanted to identify metabolic processes absent in *C. elegans* but present in other nematodes. To do this we compiled two separate lists of metabolites that are substrates of enzymes in *C. elegans* and in the other nematodes. This step was important to reduce redundancy, as more than one enzyme (EC number) can be assigned to the same step of a pathway. Next we compared the two lists and extracted those substrates missing from *C. elegans*, highlighting the enzymes that catalyse transformation of these molecules. The Enzyme Commission (EC) identifiers of these proteins were obtained through the KEGG database.

### Signal peptide prediction

Assignment of signal peptides was done using the SignalP3.0 web-interface [53] with the following parameters: organism group - eukaryotes; method - both neural networks and hidden Markov models; truncation - first 70 residues. We used three Boolean tests provided by SignalP3.0 to determine if a signal peptide was present: first ‘D’ must be true; secondly, we considered ‘Cmax’ and ‘Ymax’, if both were true then we deemed this strong evidence and weaker evidence if only one category was true. Analyses of the secreted proteomes have been carried out previously for *Nippostrongylus brasiliensis* [54] and *H. schachtii* [55]. Compared with these studies, and despite using more conservative parameters, we identified a larger number of signal peptide-containing proteins in *N. brasiliensis* (96 were identified, compared with 87 from Harcus *et al*. [54]) and *H. schachtii* (105 identified compared with 65 from Vanholme *et al*. [55]). This increase is likely to derive from more robust coding region predictions producing proteins that were more likely to contain the correct N-terminus.

### Identifying domains in nematode proteins and construction of NemDom3

NemPep3 proteins were annotated with protein domains using existing domain databases (PfamA and ProDom) and by *de novo* identification of domains in unannotated sequence.

#### PfamA domains

Domain models from PfamA version 17 were assigned in two steps. First, matches that were global with respect to the domain and local to the protein sequence were identified. Local (partial) domain matches were then selected. These second matches were only accepted if they did not overlap previous matches and occurred within 5 amino acids of termini of the protein sequences. For both global and local searches we used the hmmpfam program from the HMMer suite [56] with the gathering cut off (GA) bit score assigned to each domain as part of the Pfam curation. We removed these domain-annotated regions from NemPep3, and passed the remainder (NP3_rest) to the next step.

#### ProDom domains

The ProDom database was originally constructed using the PSI-BLAST search algorithm to identify local regions of conserved sequence in the UniProt database [35]. We filtered out those ProDom domains that matches curate PfamA entries. We used the program, *mkdom2* from the ProDom suite [57] to generate putative protein domains from NP3_rest. As EST-derived polypeptides are likely to include fragmented domains, we removed NP3_rest regions that were less than 100 residues in length. We also took advantage of the pre-filtering step of *mkdom2* to search NP3_rest with existing ProDom domain models. Novel domains were inferred using default parameters from segments remaining after identification of ProDom matches. The newly identified domains were then aligned and used to search NemPep3 to detect any domains that were present in regions excluded through length stringency cutoffs. This collection of nematode proteome-defined domains is called NemDom3.

#### Searching UniProt with novel domains

Multiple sequence alignments were constructed for each domain in NemDom3 using muscle (version 3.52) [58],[59] and used to build position specific scoring matrices (PSSM) using PSI-BLAST. The longest domain member was used as the template in each instance. The UniProt protein database was then searched against the combined library of NemDom3 novel PSSMs (one for each domain) with RPS-BLAST [60] (with an E-value cut off of 1e-5).

### NEMBASE3

NEMBASE3 is a relational database built using the PostgreSQL database manager (http://postgresql.org). It holds all the data types described above, including sequences, clustering information, consensuses derived from EST clusters, peptide predictions, protein families and protein domains. All peptides have been annotated with extensive BLAST-based similarity data, as well as quality scored functional annotation (GO, EC and KEGG identifiers) derived from GOtcha [61] and annot8r [62] analyses. The database is available through the www using custom php scripts from http://www.nematodes.org/.

## Results/Discussion

### NemPep3: inferring robust protein translations for nematode EST clusters

Coding regions for EST cluster consensuses derived from NEMBASE [12] from 37 species from the phylum Nematoda were predicted using prot4EST, yielding a total of 121,694 polypeptide sequences (Figure 1). For each species, specific codon usage tables [40] were used to reverse translate the *C. elegans* proteome, providing synthetic training-set transcriptomes (see Methods). To assess the accuracy of synthetic transcriptomes, partial datasets built for *C. elegans* [39] were translated in a similar fashion. Comparison with a complete collection of coding sequences showed only a slight reduction in prediction using synthetic transcriptomes (data not shown). Importantly, for most species the simulated training sets were more accurate than simply using the complete *C. elegans* or *C. briggsae* transcriptomes. The mean length of translation for the EST datasets (excluding the caenorhabditids) was 137 amino acids (aa) (standard deviation 65 aa), and 84% of the bases in the EST cluster consensuses contributed to translations. The regions not covered are likely to be predominantly untranslated regions, as well as regions of low-complexity sequence.

Previously, we have shown that the most accurate translations are obtained using similarity to a known protein or the prot4EST implementation of the ESTScan algorithm [38],[39]. For most nematode species, over 90% of EST cluster consensuses were translated using these two methods (Figure 1). However, three Spiruromorph species had much lower rates of translation by these methods: *Brugia malayi* (71% translated using similarity or ESTScan methods), *Onchocerca volvulus* (78%) and *Wuchereria bancrofti* (68%) (Figure 1; ‘percentage accepted’). These low rates appear to arise from two features of these data. Firstly, a relatively low proportion (∼40%) of these species' EST cluster consensuses had significant similarity to protein sequences in UniRef100 [46]. Secondly, only ∼54% of the novel sequences had compositions that matched models derived from known coding regions, simulated transcriptomes, or, in the case of *B. malayi* where a first pass annotation of the whole genome sequence is available [63], an extensive transcriptome dataset.

Our inability to derive high quality translations for a significant number of clusters from these taxa could be due to a major biological difference and to the quality of the training set used or to the quality of the sequence data. Other species that had similarly low proportions of sequence similarity matches, had higher rates of compositionally-identified coding regions (e.g. *Trichuris vulpis* with 80% of the novel sequences translated by ESTScan and *Meloidogyne javanica* with 97%). The addition of a 12,000-transcript, orthologous training set [63] did not improve the proportion of *B. malayi* cluster consensuses that yielded a translation. For these three problem species, we noted that singleton cluster consensuses were much less likely to be robustly translated, but these species did not have an excess of singletons compared to the other nematodes. The proportions of ESTs lacking detectable coding regions were compared between the source cDNA libraries. Of 25 *B. malayi* libraries, five were significantly enriched for ESTs not translated (G-statistic = 682; p≪0.001). Two libraries from the eight available for *O. volvulus* and two for *W. bancrofti* were also shown to contain an excess of ESTs without a coding region. Strikingly, 93% of the untranslatable sequences from *B. malayi* came from the highlighted five libraries, while the *O. volvulus* and *W. bancrofti* libraries accounted for around 30% of each species suspect contigs. We conclude that some of the unique features of the three species' data derive from the relative quality of some cDNA libraries sampled.

To ensure that subsequent analyses were performed on the most accurate collection of polypeptides, we excluded EST cluster consensuses that could not be translated with either the sequence similarity or ESTScan components of prot4EST. Addition of the proteomes from the fully-sequenced *C. elegans* and *C. briggsae* yielded a high quality dataset (NemPep3). The current release of NemPep, version 3, includes 154,501 polypeptide sequences (Figure 1), with a mean length of 220 amino acids. NemPep3 is available for download from NEMBASE3 (http://www.nematodes.org/nembase3/).

### Islands in nematode protein space: protein families

We used TRIBE-MCL [50] to derive putative protein families (NemFam3) from NemPep3. These families were compared to proteins from the UniProt database [46] to identify overlap with previously defined protein families. The results of the clustering algorithm, MCL, can be tuned with an Inflation parameter. In the context of protein clusters, this value determines how tight, or strict, the clustering is (see Methods). No single parameter set for TRIBE-MCL can be used to accurately identify all (or even most) families and so we generated independent estimates at five different Inflation values. To simplify analyses presented here, we have examined in detail the 65,179 protein families generated using an Inflation value of 3.0, the default used for the TRIBE-MCL database [64].

**Figure 2:**
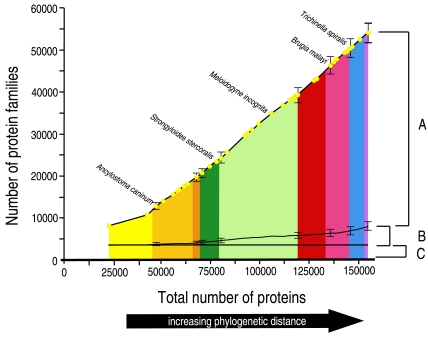
Protein family discovery in the phylum Nematoda. Nematode protein families (NemFam3) were generated using Markov flow clustering [50] with a range of Inflation parameters. The bars show the extreme number of protein families considering different Inflation parameters. Here we analyse families defined with an Inflation parameter of 3.0. A collector's curve was derived as described in Materials and Methods. Yellow circles indicate the cumulative counts of proteins (x-axis) and unique families (y-axis) as each species was added. The upper black line follows the cumulative number of protein families identified as each new species was included. For example, the 4,368 protein sequences from *A. caninum* included 1,200 NemFam3 families not present in the *Caenorhabditis* proteomes. The middle black line tracks the cumulative number of NemFam3 protein family models that identify representatives in non-nematodes, and the bottom line shows the number of NemFam3 protein family models that were present in *C. elegans* and in species from other (non-nematode) phyla. Region A protein families were restricted to nematodes (given current databases), while region B families have been lost in *C. elegans* or gained in specific nematode lineages (loss/gain candidates) and are shared with non-nematode taxa. Region C protein families are shared between *C. elegans*, other nematodes and non-nematode species.

**Figure 3:**
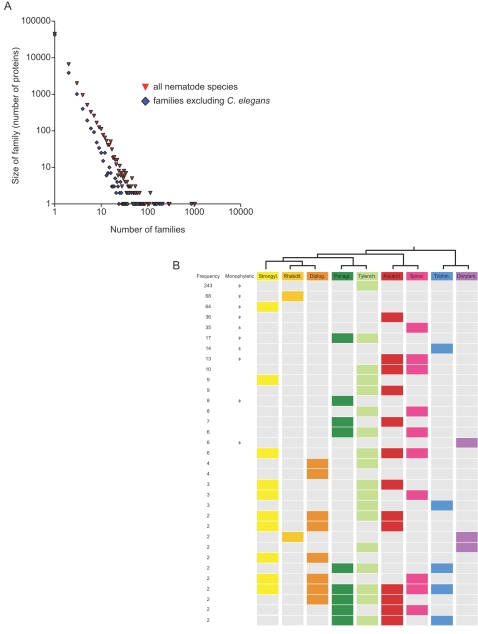
Nematode-restricted protein families. (A) Distribution of protein family size can be described by a power law, with a large number of small families and the number of families decreasing as their size increases. Removing *C. elegans-*containing families reduced the total number of families, but the power law distribution persisted. (B) Many protein families had restricted taxonomic distribution within Nematoda. For all protein families with at least five non-*C. elegans* members, the systematic affinities of the contributing species were compared. Proteins families were identified that were restricted to each of the taxonomic families represented in the analysis, and to higher-level taxonomic groups (e.g. the Spirurina which includes Ascaridomorpha and Spiruromorpha). For example, 243 protein families were restricted to the Tylenchomorpha One species from a taxonomic family needed to be represented in the protein family for inclusion in the figure.

Despite having a large sample (37 species and over 150,000 individual sequences) we found no evidence of having exhausted the diversity of nematode ‘protein space’. There was a near-linear increase in the number of protein families identified with addition of sequences and species (Figure 2). This finding is congruent with that of Parkinson *et al*. (2004b) but here we have used a rigorous protein family definition schema rather than simply BLAST matches. Analyses of complete prokaryote proteomes also show an increase in the number of novel proteins as further species are sequenced [65], although as a proportion of all prokaryote proteins the number of novel proteins is decreasing [66]. This trend is not apparent in the nematode dataset (Figure 2). The distribution of size of the NemFam3 protein families can be described by a power law, matching that of many protein family databases (Figure 3a) [67].

We identified protein families that were restricted to all levels of nematode taxonomy, from species-specific to phylum-specific (Figure 3b). By comparing NemFam3 families to proteins from non-nematode species, we divided them into three classes: NemFam3 families that were unique to the Nematoda (region A of Figure 2); NemFam3 families that were not found in *C. elegans* but did have homologues in other phyla (region B); and NemFam3 families that included *C. elegans* members and had homologues in other phyla (region C). Region C presumably encompasses proteins with core metabolic functions shared with other phyla.

### Gene loss in *C. elegans* (Figure 1, region B)

Gene loss is a common feature of genome evolution [68]–[70]. Gene gain by horizontal gene transfer is common in non-eukaryotes, but its role in eukaryotes, and particularly in metazoans, is still controversial [71]–[73]. Gene loss in *C. elegans* has been reported previously [6], [74]–[76]. For example, orthologues of the Hox genes Antennapedia and Hox3 are absent in *C. elegans* but present in *B. malayi* and other invertebrates [76]. Comparison of *C. elegans* and *C. briggsae* [6] identified a large number of proteins in each species that did not have an orthologue in the other. Using NemPep3 and the UniProt database (release 5) reduces the number of orphan proteins in *C. elegans* from 2,108 to 1,846 and from 2,141 to 1,961 in *C. briggsae*. Comparison with proteomes from additional *Caenorhabditis* sp. genomes currently being sequenced will clarify the patterns of gene gain and loss in this lineage.

We identified 4,864 protein families (containing 6,903 proteins) that had significant sequence similarity to proteins from outside the Nematoda but that contained no *C. elegans* representatives (‘loss/gain candidates’). To investigate the effect of using partial sequences, we compared loss/gain candidate EST cluster consensuses to the *C. elegans* genome. Thirty-nine loss/gain candidate families (92 sequences) could be aligned to the genome (using the program BLAT [77]) and overlapped an annotated coding sequence: the failure of TRIBE-MCL to group the *C. elegans* proteins with their loss/gain candidate matches was because their BLAST alignments had a low similarity score. Three loss/gain candidate families (eight proteins) matched regions of the *C. elegans* genome that were not part of a coding region: these may correspond to valid but unannotated genes in *C. elegans*. Thus the majority of loss/gain candidate families are absent from *C. elegans*.

**Figure 4:**
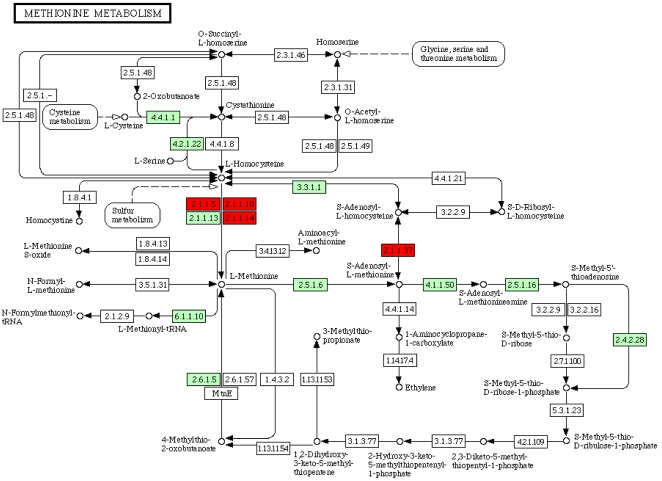
Methionine metabolism in nematodes. Cytosine-5′-methyltransferase (EC 2.1.1.37) is not present in *C. elegans* but has been detected in four phylogenetically divergent nematode species, suggesting that it may be widespread throughout the phylum and lost in the *Caenorhabditis* lineage. Enzymes found in *C. elegans* are green, those present in other nematodes but absent in *C. elegans* are red. Three further enzymes were identified as possible candidates for gene loss in *C. elegans.* Betaine-homocysteine S-methyltransferase (EC 2.1.1.5), homocysteine S-methyltransferase (EC 2.1.1.10) and 5-methyltetrahydropteroyltriglutamate–homocysteine methyltransferase (EC2.1.1.14) were found in one, seven and four nematode species, respectively, but not in *C. elegans*. The latter two enzymes have not previously been reported in metazoans and their identification in plant-parasitic nematodes may be a result of horizontal gene transfer.

Gene Ontology (GO) annotation of the loss/gain candidate families showed that a large number are involved in metabolism. One hundred and fourteen individual Enzyme Commission (EC) classifications could be assigned to 240 families (a full list of these annotations is available in Table S1). Some of these putative functions complemented gaps in the metabolic map of *C. elegans*. For example, *C. elegans* lacks a canonical DNA methylation pathway enzyme, cytosine-5′-methyltransferase [78]. Homologues of cytosine-5′-methyltransferase were identified in *Ostertagia ostertagi*, *Teladorsagia. circumcincta* and *Xiphinema index* (Figure 4), and a homologue has also been identified in *Pristionchus pacificus* [79]. It will be informative to examine additional nematode genomes for the features of DNA methylation and thus identify when, and perhaps why, this core regulatory mechanism was lost.

### Gene gain by putative horizontal transfer

While the above examples reveal the process of gene loss, we also identified putative gain of genes by horizontal transfer from other organisms (Table 1). Plant-parasitic nematodes modulate their host's metabolism and induce development of feeding sites (for example induction of syncytia by cyst nematodes, and of giant cells by root-knot nematodes). These modifications involve the secretion by the nematode of exoenzymes such as pectinases, proteinases and cellulases (reviewed by Vanholme and colleagues [80]). Putative effectors have been identified using directed cloning of nematode secretory gland products, including beta-1,4-endoglucanases from *Globodera rostochiensis* [81], *Heterodera schachtii* [82] and *Meloidogyne incognita* [83]. Analyses of plant-parasitic EST data also identified beta-1,4-endoglucanase, beta-1,4-xylanases [84] and pectate lyases [85]. We identified two *Meloidogyne* orthologues (*M. javanica* and *M. hapla*) of a polygalacturonase previously reported from *M. incognita* [83]. Beta-1,4-endoglucanases were identified in seven species, including *Pratylenchus vulnus*. The enzyme's presence across most Tylenchid genera studied (missing in the small *Rhadopholus similis* dataset) suggests that the acquisition of this endoglucanase gene occurred in an ancient tylench ancestor.

**Table 1:** Plant-like enzymes identified in nematode proteomes.

EC number	Enzyme Name	Enzyme Description	Nematode species with this annotation *
1.14.11.23	flavonol synthase	synthesises quercetin, a nematotoxic isoflavonoid [101]	*X. index*
2.4.1.228	scopoletin glucosyltransferase	activates scopoletin to scopolin, which is involved in pathogen responses and lesion formation	*X. index*
3.1.1.78	polyneuridine-aldehyde esterase	synthesis of the skeleton of sarpagan (an alkaloid and thus likely defence metabolite)	*M. incognita*
3.2.1.15	polygalacturonase	pectinase; hydrolysis of 1,4-alpha-D-galactosiduronic linkages in pectate and other galacturonans	*M. javanica*, *M. hapla*
3.2.1.67	galacturan 1,4-alpha-galacturonidase	cell wall breakdown pectinase; exopolygalacturonase	*M. arenaria*, *M. incognita*
3.2.1.2	beta-amylase	starch catabolism	*H. glycines*
3.2.1.4	cellulase (several forms)	catabolism of plant cell wall celluloses	*G. pallida*, *G. rostochiensis*, *H. glycines*, *H. schachtii*, *M. arenaria*, *M. incognita*, *M. hapla*, *M. incognita*, *M. javanica*, *P. vulnus*

*: Protein identifiers are available in Table S5.

We identified seven additional protein families from plant parasitic nematodes that are similar to enzymes found in plants but not previously identified in non-nematode metazoans. The activities that may be carried out by these genes fall into two classes. Four genes, all from Tylenchomorpha, are enzymes that catabolise plant cell wall or starch carbohydrates (polygalacturonase, beta-amylase and cellulase), and may mediate parasite modification or digestion of the root cell walls. Three genes, from the dorylaim *X. index* and the tylenchomorph *M. incognita*, encode activities that could modify plant signaling or second metabolites (flavonol synthase, scopoletin glucosyltransferase and polyneuridine-aldehyde esterase), and may represent ‘anti-immunity’ mediators secreted by the parasite in order to subvert the necrotic or other responses of the host.

### Gene gain by *de novo* evolution (Figure 1, region A)

Another mechanism of “gene gain” is *de novo* evolution of functional proteins. While it is clear that this mechanism has been active on the scale of phyla and kingdoms, its ongoing role in genome evolution is unclear [86]. We identified 56,407 protein families (including 94,343 proteins) restricted to nematodes (NR families). Analyses of novel proteins in other species have shown that they are characterized by a significant reduction in average length compared to proteins with homologues in other taxa [65]. However, the average length of the NR family proteins (200 aa) is only slightly shorter than those with homologues elsewhere (220 aa). It might be expected that novel genes would be expressed at low levels, and that they might thus be indistinguishable from aberrant transcripts from non-coding regions of the genome. Over 80% of the NR families contained an EST-derived sequence; not restricted to the caenorhabditids. Of these 69% were derived from a single EST (data not shown). For loss-gain candidate protein families, 68% were derived from a single EST, while of families with matches in *C. elegans* and elsewhere, only 35% were derived from single ESTs. Thus, while the NR family sequences are expressed at low levels compared to core nematode genes, their expression levels are comparable to those of genes with wide phylogenetic distribution.

We analyzed further the 2,098 NR families with at least five members. The number of NR families restricted to each taxonomic family or species correlated well with the depth of sequencing for each taxon (Table S2). We note that despite cogent evidence for gene loss in the caenorhabditids [74]–[76], many NR families with a disjoint distribution in Nematoda are likely to be present in additional species, but as yet unsampled by ESTs. For example, 388 protein families (2,985 proteins) were restricted to the complete proteomes of the caenorhabditids (Family Rhabditoidea). The lack of homologues in other nematodes is likely to result in part from the depth of EST sampling, as only 1,385 (46%) of these proteins had corresponding *C. elegans* ESTs (out of 346,064 EST sequences).

All nine nematode taxonomic families in this study had taxon-restricted protein families. For example, of 35 protein families that were restricted to Spiruromorpha, only three were species-specific (one restricted to *B. malayi* and two to *O. volvulus*; data not shown). Fourteen of the spiruromorph protein families occurred in four species and one (NemFam3 family 3.0_3062) contained all five species (whose multiple sequence alignment is shown in Figure S1). Many (630) NR protein families with at least five members did not contain a protein from the complete proteome of *C. elegans*.

The processes of ‘gene invention’ (and high rate of protein evolution) are ongoing in Nematoda. Indeed, the preponderance of apparently species-specific proteins is just what we would predict from this process, given the pull towards new functions, and thus may not be simply due to lack of representation in EST data. However, compared with our previous analysis [11], many sequences once thought to be species-specific now have inferred nodes of origin deeper in the nematode phylogeny, and we would expect this trend to continue as additional data are collected.

### Do nematode secretomes evolve novelty faster?

**Figure 5:**
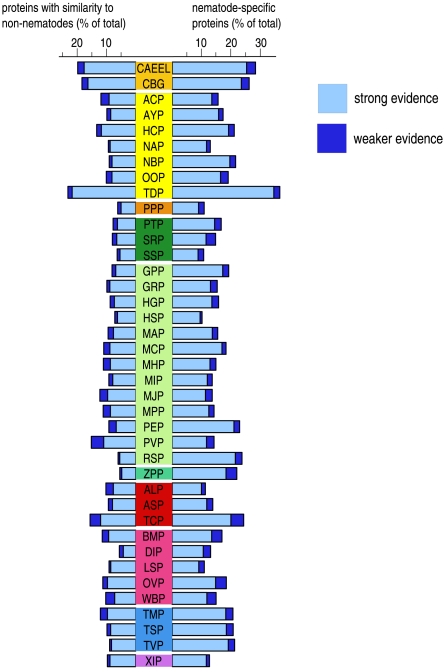
Signal peptides in nematode proteomes in NemPep3. Signal peptides were predicted in NemPep3 using SignalP [53]. For each species the proportion of signal peptide-containing proteins is given. There is a significant increase in the proportion of novel nematode proteins containing signal peptides relative to proteins with homologues in other phylum (p<0.0001; t = 10.53230; df = 38; paired t-test with data arcsin transformed).

It has been hypothesized that the secreted subset of parasitic nematode proteomes may be especially enriched in novel proteins, through rapid evolution to perform novel functions such as interactions with the host and other environmental challenges [54],[55]. The protein families restricted to the nematodes were significantly enriched for signal peptides (19%) compared to those that had homologues in other phyla (12%) (Figure 5). Within the class of nematode protein families that did have homologues in other phyla (non-NR), 2,490 proteins (28%) were predicted to have signal peptides. Surprisingly, aligning these signal peptide-containing nematode proteins to homologues from other phyla revealed that 1,883 nematode proteins (from 856 NemFam3 families, both NR and non-NR) appear to have gained an N-terminal signal peptide. For two thirds of these protein families, *C. elegans* and *C. briggsae* proteins do not contain a signal peptide, suggesting that the acquisition of a signal peptide did not occur in the caenorhabditid lineage. The *T. circumcincta* proteome was the most enriched with signal peptides in both nematode-restricted and shared proteins. Mapping these *T. circumcincta* proteins onto NR families identified 48 strongylomorph-restricted families where signal peptide-containing proteins predominated. Despite the incomplete sampling of nematode protein space it is likely that many of these protein families are involved in specializations of the parasitic mode of life in strongylids.

### NemDom3: Domain analysis of nematode proteomes

Domains are the basic functional and structural units of proteins and, while primary sequence diversity is expected to be huge, the diversity of domains has been predicted to be rather small [87],[88]. As novel genes are being evolved in nematodes, we predicted that there might be *de novo* or accelerated evolution of protein domains. Identification of protein domains typically involves comparing sequences to a library of protein domain alignments [32],[33],[35]. These alignments are characterized either as hidden Markov models (HMM) or position-specific scoring matrices (PSSM). Such an approach is well suited for full-length sequences, where a match, global (i.e. full-length) with respect to the domain, is usually considered necessary. However, proteomes derived from EST projects contain incomplete sequences, where only part of the domain is present making these global searches problematic. In particular it is difficult to robustly recognize domains that extend over the termini of partial translations. We devised a heuristic approach to assigning domain presence, based on different scoring thresholds available for domain models, in order to return a high coverage of domain annotation while keeping number of false positives to a minimum (see Materials and Methods).

The resulting nematode domain classification (NemDom3) contained 39,944 unique domains (Table 2) of which 2,593 were from PfamA and 10,684 from ProDom. The majority of these domains were derived from the complete caenorhabditid genomes, but more than half were found in the EST-derived proteome.

**Table 2:** The domain content of nematode proteomes.

	Domain definition source			
	PfamA	ProDom	NemDom3 Novels	All Classes
Number of unique domains	2,593	10,684	23,317	36,594
excluding caenorhabditid proteins	2,300	5,550	10,833	18,683
not present in caenorhabditids^a^	214	807	7,660	8,681
Total number of domain instances	68,302	95,904	69,301	233,507
Total number of proteins with one instance of domain class^b^	52,092	44,538	110,540	131,502
coverage (percent of amino acids)	22.7%	25.2%	36.9%	84.9%
coverage excluding caenorhabditid proteins (percent of amino acids)	21.2%	16.8%	20.6%	58.6%
Number of species-specific domains	487	3,318	5,560	9,365
excluding caenorhabditids^c^	168	578	2,689	3,435
Number of taxonomically restricted domains^d^	394	5,800	19,221	31,274
number of domains restricted to Strongyloidea	12	51	878	941
number of domains restricted to Rhabditoidea	293	5,134	12,484	17,911
number of domains restricted to Panagrolaimomorpha	6	35	383	424
number of domains restricted to Tylenchomorpha	34	190	3,777	4,001
number of domains restricted to Ascaridomorpha	3	30	555	588
number of domains restricted to Spiruromorpha	15	201	756	972
number of domains restricted to Trichinellida	7	53	252	312
number of domains restricted to Dorylaimida	24	106	136	266

a any domain that occurred in *C. elegans* or *C. briggsae* is ignored.

b proteins are only counted once.

c excludes proteins from *C. elegans* and *C. briggsae*. The domain family may occur in these species, but must also be present in another species to be counted.

d the taxon specificity is with respect to the nematode taxonomic family. Domains included annotated as “family-specific” here may also be found in other phyla. This is particularly true for PfamA and ProDom domains.

Previously, 348 PfamA domains had been identified in non-caenorhabditid nematodes. We found 2,300 PfamA domain matches in the EST-derived proteomes of which 214 domains (increased from thirteen) were absent in *C. elegans* and *C. briggsae*. All but eight of these domains were exclusive to protein sequences that we had already identified as loss/gain candidates (described above), including those restricted to plant-parasites: cellulase (PF00150) and pectate lyase (PF03211). Of the eight domains identified in protein families that include *Caenorhabditis* sp. members, two of these, domains associated with the ribosomal large subunit protein 6 (PF03868) and NADH:ubiqunione oxidoreductase (PF08122), have been reported in *C. elegans* [89]. However their sequences have been so diverged from the domain model as not to be recognized.

Seventy-seven PfamA domains were found only in nematodes, with six found in species other than exclusive to *C. elegans* or *C. briggsae* (Table S3). With the exception of the abundant larval transcript (ALT) domain (PF05535), all nematode-restricted (NR) domains were first identified in *C. elegans* [90]–[93]. Surprisingly, we were able to expand the species-distribution in only 24 of the 77 domains. It is possible that the remaining NR domains are restricted to the caenorhabditid lineage. However, it is more likely that many, if not most, are present in other nematode species, but were not yet represented in EST data, or were not recognized by domain models that were too constrained. Inspection of the multiple sequence alignments of caenorhabditid-specific NR domains revealed often extremely high levels of identity. These alignments may generate hidden Markov models (HMMs) that cannot identify more divergent members. To illustrate this, we returned to the ALT domain (PF05535), which was, expectedly, identified in proteins from filarial species, but the searches did not find the known instance in *C. elegans* [90],[91]. Using the Pfam alignment for this domain (based on five filarial sequences), we constructed a PSSM and performed a RPS-BLAST search. This identified ALT domains in *C. elegans* as well as predicted proteins from *Ascaris suum* and *A. lumbricoides*.

### Novel domains in nematode proteomes

We defined over 23,000 protein domains seemingly unique to nematodes. Nearly half of these are found in non-caenorhabditid species. Many of these new domains are found as part of multi-domain architectures, with 15,152 (65%) present with at least one different domain (all classes) and 6,625 associated with a PfamA domain. Profile searches with these novel domains (see Methods) identified 3,694 domains that matched non-nematode UniProt proteins. The most common distribution of these domains was the 270 domains found throughout the Ecdysozoa. However many domains had disjoint distributions, such as the 56 novel domains apparently exclusive to the nematodes and Viridplantae. Ten of these domains were found in plant-parasite nematode species (Table 3). The presence of putative homologues for three of these domains in *C. elegans* confuses of the issue of their origin. The absence of these domains in other metazoans suggests that they were either acquired through horizontal gene transfer or diverged from an ancestral nematode domain. Convergent evolution has been reported previously in nematodes [94],[95].

**Table 3:** Novel NemDom3 domains also identified in plants (Viridiplantae).

NemDom3 identifier	domain length (amino acids)	Species*	Present in *C. elegans*	plant species	UniProt accessions	functional annotation
ND_n0000006890	42	*M. arenaria*	yes	*Oryza sativa*	Q5Z9Q3, Q6MWB4, Q7XLT3	wall-associated receptor kinase-like 21 precursor
		*M. incognita*		*Prunus persica*	Q6DU55	
				*Phaseolus vulgaris*	Q94KF4, Q94KF5	
				*Arabidopsis thaliana*	Q67YK2, Q8GYF5, Q9LDZ5, Q9LFL1, Q9FL01	
ND_n0000004827	42	*G. pallida*	no	*Zea mays*	Q5EUC0	thiol oxidoreductase
		*H. glycines*				
ND_n0000010444	56	*G. pallida*	yes	*Lycopersicon esculentum*	GSHB_LYCES	glutathione synthetase
		*M. arenaria*				
		*M. chitwoodi*				
ND_n0000022177	83	*M.arenaria*	no	*Oryza sativa*	Q40625, Q2QVD7	BZIP transcription factor family
		*M. incognita*				
ND_n0000005472	41	*H. glycines*	yes	*Arabidopsis thaliana*	GST16_ARATH, Q1WW15	glutathione S-transferase
		*G. rostochiensis*		*Solanum commersonii*	O22330	
		*M. hapla*		*Capsicum chinense*	Q5DUH0	
				*Brassica juncea*	Q7XZT0, Q7XZT2, Q7XZT3	
				*Cucurbita maxima*	Q8GT24	
				*Euphorbia esula*	Q9M533	
				*Oryza sativa*	Q56XF1, Q93WM2, GSTH2_ORYSA	
ND_n0000017177	94	*M. arenaria*	no	*Arabidopsis thaliana*	Q9ZQ31	hypothetical protein
		*M. chitwoodi*				
		*M. incognita*				
		*M. paranaensis*				
ND_n0000021399	51	*M. hapla*	no	*Arabidopsis thaliana*	Q9FGC2	DNA helicase-like
		*M. javanica*				
ND_N0000004924	39	*H. schachtii*	no	*Triticum aestivum*	Q84VR8	chimaeric SDH2-RPS14 protein
		*M. arenaria*				

*: Protein identifiers are available in Table S6.

Are these domains real, conserved units? Of the 1,652 novel domains that were exclusive to the Spiruromorpha, 824 were found in at least two species of this taxon (Table S4). Of this latter set, 435 are associated with Pfam or ProDom domains. Being shared across a number of species suggests that these domains are likely to be functional. Hints as to their function may be derived from their associations with previously characterized domains, and from other high-volume datasets such as genome-wide RNAi screens and protein-protein interaction maps.

### Presenting nematode protein space: NEMBASE3

The resources we have generated (NemPep3, NemFam3 and NemDom3) are presented in an interactive interface in the NEMBASE database at http://www.nematodes.org/ [12]. Release 3.1 of NEMBASE3 contains 128,709 EST clusters, and 31,461,090 annotations from 37 nematode species. Data in NEMBASE3 can be searched for individual ESTs, clusters, stage-specific and overall expression levels (derived from EST counts), protein translations, domains, and families. Functional annotations (Gene Ontology categories, Enzyme Commission numbers, metabolic pathways and best BLAST matches) are also available.

### Conclusion

ESTs are typically used to annotate newly assembled genomes or provide snapshots of transcriptomes. Here we have shown that by both clustering (creating a reference sequence or unigene set) and careful translation, they can yield high quality partial proteome data. Importantly, the additional effort expended in deriving high quality translations is repaid in the increase in mean lengths of derived proteins, and in the increase in ascribable annotations. This is particularly evident in the correct identification of extended 5′ open reading frames from regions of lower quality EST sequence, and thus an enhanced ability to identify signal peptides (Figure 5). Issues of lack of relevant training data for model-based identification of open reading frames in neglected species can be overcome by bootstrapping BLAST-identified open reading frames to generate codon usage tables and synthetic proteomes.

Comparison to the complete proteomes derived from genome sequence emphasizes the partial nature of EST-derived proteomes. Many genes with core roles in metabolism or signaling pathways are absent from the nematode partial proteomes, but this is likely to be due to lack of evidence rather than true loss. The EST-derived partial genomes systematically lack, or have very reduced, representation of some classes of genes. Thus, while the seven transmembrane helix class of odorant receptor gene is the most abundant gene family in *C. elegans*, homologues are conspicuously lacking from EST-derived proteomes. Indeed, even within the large *C. elegans* EST collection, no transcript is assigned to an odorant receptor.

However, by comparison to complete genomes, EST-derived proteomes can be used to highlight gene loss events in fully sequenced species. Using this methodology we identified a significant number of gene families (4,800) absent in *C. elegans* but present in other nematodes and in other phyla. Some of these genes have likely been lost from *C. elegans*, as they have wide representation in other nematodes, and in non-nematode phyla. The loss of developmental pathway genes such as members of the Hox cluster, and of hedgehog homologues, has been associated with the evolution of a strict, lineage-based developmental control system in *C. elegans*. We identified additional losses of this type, including the loss of key DNA methylation genes.

Other candidates for loss in *C. elegans* had a distinct pattern of presence in other phyla: they were found in only a restricted subset of nematode species and also in a disjoint group of organisms (such as plants or bacteria). The limited occurrence of these genes is perhaps best explained by horizontal transfer from a host plant or other closely associated genome into the nematode genome. Notably, the proteomes of the plant parasitic Tylenchina contained genes of apparent plant or rhizosphere bacterial origins. Our analysis pushes the event(s) of acquisition of these classes of genes deeper into the tylenchine phylogeny, supporting the hypothesis that they may have been a key innovation leading to plant parasitism in the whole group.

Another deeply sampled taxon was the medically important Spiruromopha. We have identified 35 protein families that are restricted to this lineage. Importantly, fourteen families had membership in at least four of the five species surveyed. These groups are ideal candidates for functional genomic and reverse genetic technologies that could reveal their function and importance to the survival of these parasitic worms, and thus whether they are possible targets for a next generation of anthelminthic drugs.

Cross-comparison of the *C. elegans* and *C. briggsae* proteomes identified ∼10% of unique genes in each species. Throwing the draft B. malayi genome into the mix, revealed ∼40% of its proteins did not share homology to *C. elegans*, *C. briggsae* nor *Drosophila melanogaster* [63]. Adding partial proteomes from 37 additional nematode species reduced the number of private genes to ∼8% in each species. While we expect this proportion to decline as nematode EST sequencing continues, along with the release of genomes, we expect that each fully sequenced genomes has a significant complement of novel genes that have arisen since they last shared a common ancestor, less than 100 million years ago [6],[96],[97]. If this pattern is true of all the >1 million predicted nematode species, then ‘nematode protein space’, the portion of possible sequence structures actually occupied by nematode proteins, is likely to be huge. Our analyses suggest that nematode protein space is huge, and that it is likely that our survey has merely scraped its surface. Indeed, some closely-related species, particularly within the Tylenchina, have an even higher proportion of private genes. This pattern is observed in all-against-all BLAST comparisons, in *de novo* protein family definition, and in derivation of novel domains. Most Nemfam3 families and NemDom3 domains are apparently private to Nematoda, and many have restricted phylogenetic distributions within the phylum.

This finding contrasts with that emerging from whole genome analysis within Mammalia, where comparison of the predicted proteomes of eutherian (human) and metatherian (opossum) identified only 624 genes private to opossum and ∼500 to human (about 2.5% of the predicted gene complement of each species), despite ∼180 million years of divergence [98]. However, comparisons of the predicted genes of the osteichthean *Oryzias latipes* (medaka) to those of other fish such as *Tetraodon nigroviridis*, with which medaka last shared a common ancestor ∼190 million years ago, identified 2936 genes unique to medaka, ∼15% of the total gene count [99]. Similarly, cross-comparison of the *D. melanogaster* (fruit fly), *Anopheles gambiae* and *Aedes aegypti* (mosquito) proteomes identified 2924 (22%) *A. gambiae* and 4181 (27%) *A. aegypti* genes that were private to each species [100]. The mosquitoes are estimated to have diverged ∼140–200 million years ago. Thus the finding of high rates of novel gene evolution in the Nematoda may reflect a common pattern in Metazoa, with vertebrate taxa having a reduced rate.

The identification of this level of protein novelty also challenges estimates of the total number of different protein families, and of the number of different possible domains, in all protein space. Even if our estimates of domain diversity are inflated through difficulties engendered by the use of partial proteome sequences, we have identified as many different domains in Nematoda as have been predicted in the rest of Metazoa to date. Additional meta-analyses of other major non-vertebrate groups, such as Arthropoda and Annelida, are sorely needed to explore the generality of these findings.

## Supporting Information

Figure S1 Alignment of five species spiruromorph family
(0.36 MB EPS)

Table S1 Metabolism table: loss/gain
(0.03 MB XLS)

Table S2 Family and species-specific protein families
(0.42 MB PDF)

Table S3 71 PfamA nematode-restricted domains with nematode distributions
(0.01 MB TXT)

Table S4 Species distribution of novel domains
(0.33 MB PDF)

Table S5 Plant-like enzymes identified in nematode proteomes (includes protein identifiers)
(0.33 MB PDF)

Table S6 Novel NemDom3 domains also identified in plants (includes protein identifiers)
(0.45 MB PDF)

Alternative Language Abstract S1 Translation of the abstract into German by Ralf Schmid. Übersetzung der Zusammenfassung ins Deutsche von Ralf Schmid.
(0.03 MB DOC)

Alternative Language Abstract S2 Translation of the abstract into French by Douglas Finney. Traduction du résumé en français par Douglas Finney.
(0.03 MB DOC)
