# Supplementary material for: On the Extent and Origins of Genic Novelty in the Phylum Nematoda
Source: PLoS Negl Trop Dis. 2008 Jul 2;2(7):e258. doi: 10.1371/journal.pntd.0000258 (PMC2432500; doi:10.1371/journal.pntd.0000258)
Supplement: Table S2 — Family and species-specific protein families (0.42 MB PDF) [file pntd.0000258.s003.pdf]

Table B – Family and Species-specific protein families

Taxonomic family	species	Proteins Available	Proteins restricted to Nematoda	Family restricted protein families	species-specific protein families
Spiruromorpha	5	15896	9621	35	3
Diplogasteromorpha	1	4229	1221	4	4
Pangrolaimomorpha	3	10421	5478	8	6
Ascaridomorpha	3	10852	7430	36	13
Strongyloidea	7	20713	12554	64	29
Dorylaimida	1	4335	1860	6	6
Trichinellida	3	4861	3546	14	5
Rhabditoidea	2	62672	26538	388	111
Tylenchomorpha	13	55465	26150	243	21

The phylogenetic distribution of taxonomically-restricted protein families. A protein family needed to contain at least five proteins to be included. In contrast to figure 4b, *C. elegans* containing protein families were used in this table. Counts of species-specific protein families consider only the species covered by that row's taxonomic family. While, a large proportion of taxonomically-restricted protein families are species-specific, many contain proteins from more than one species.
