# Supplementary material for: On the Extent and Origins of Genic Novelty in the Phylum Nematoda
Source: PLoS Negl Trop Dis. 2008 Jul 2;2(7):e258. doi: 10.1371/journal.pntd.0000258 (PMC2432500; doi:10.1371/journal.pntd.0000258)
Supplement: Table S4 — Species distribution of novel domains (0.33 MB PDF) [file pntd.0000258.s005.pdf]

Table D – Novel protein domains restricted to monophyletic taxa

Taxon-specific	***number of species with protein domain***										
	1+	***2+***	***3+***	***4+***	***5+***	***6+***	***7+***	***8+***	***9+***	***10+***	***number of protein domain groups***
Ascaridomorpha	794	293	13								
Caenorhabditida	14,785	12,302									
Spiruromorpha	1,230	824	175	39	2						
Strongylida	1,652	949	204	60	19	6	2				
Trichinellida	359	128	7								
Tylenchomorpha	5,464	3,885	1,108	401	164	48	18	8	2	1	
