# Supplementary material for: On the Extent and Origins of Genic Novelty in the Phylum Nematoda
Source: PLoS Negl Trop Dis. 2008 Jul 2;2(7):e258. doi: 10.1371/journal.pntd.0000258 (PMC2432500; doi:10.1371/journal.pntd.0000258)
Supplement: Table S5 — Plant-like enzymes identified in nematode proteomes (includes protein identifiers) (0.33 MB PDF) [file pntd.0000258.s006.pdf]

Table E - Plant-like enzymes identified in nematode proteomes

EC number	*Enzyme Name*	*Enzyme Description*	*NemPep3 proteins with this annotation* *
1.14.11.23	flavonol synthase	synthesises quercetin, a nematotoxic isoflavonoid (Osman and Viglierchio 1988)	XIP02339_1
2.4.1.228	scopoletin glucosyltransferase	activates scopoletin to scopolin, which is involved in pathogen responses and lesion formation	XIP03415_1
3.1.1.78	polyneuridine-aldehyde esterase	synthesis of the skeleton of sarpagan (an alkaloid and thus likely defence metabolite)	MIP07018_1
3.2.1.15	polygalacturonase	pectinase; hydrolysis of 1,4-alpha-D-galactosiduronic linkages in pectate and other galacturonans	MJP01724_1, MHP05484_2
3.2.1.67	galacturan 1,4-alpha-galacturonidase	cell wall breakdown pectinase; exopolygalacturonase	MAP00565_1, MIP04638_1
3.2.1.2	beta-amylase	starch catabolism	HGP00188_1
3.2.1.4	cellulase (several forms)	catabolism of plant cell wall celluloses	GPP00099_1, GPP00676_1, P000664_1, GRP01255_1, HGP06814_1, HGP03686_2, HGP07086_2, HGP07404_2, HGP06600_1, HGP06861_2, HSP00667_1, HSP00991_1, HSP01296_1, HSP00352_1, MAP00224_1, MAP00702_3, MAP00702_2, MCP01234_1, MCP05954_1, MCP00574_2, MCP00574_1, MCP03969_1, MCP00966_2, MHP03294_2, MHP04018_2, MHP03286_2, MHP11140_1, MHP12261_1, MHP01099_1, MHP04209_1, MHP11913_1, MHP04129_1, MHP09658_1, MIP00115_1, MIP00028_2, MIP02244_1, MIP00867_1, MJP04028_1, MJP03779_1, MJP04327_1, PVP00108_1

* Underlined protein identifiers have predicted signal peptides.
