# Supplementary material for: On the Extent and Origins of Genic Novelty in the Phylum Nematoda
Source: PLoS Negl Trop Dis. 2008 Jul 2;2(7):e258. doi: 10.1371/journal.pntd.0000258 (PMC2432500; doi:10.1371/journal.pntd.0000258)
Supplement: Table S6 — Novel NemDom3 domains also identified in plants (includes protein identifiers) (0.45 MB PDF) [file pntd.0000258.s007.pdf]

Table F - Novel NemDom3 domains also identified in plants (Viridiplantae)

NemDom3 identifier	domain length (amino acids)	NemPep3 sequences *	Present in *C. elegans*	Plant species	UniProt accessions	functional annotation
ND_n0000006890	42	MIP03120_1<br>MAP00264_1	yes	*Oryza sativa*<br>*Prunus persica*<br>*Phaseolus vulgaris*<br>*Arabidopsis thaliana*	Q5Z9Q3, Q6MWB4, Q7XLT3<br>Q6DU55<br>Q94KF4, Q94KF5<br>Q67YK2, Q8GYF5, Q9LDZ5, Q9LFL1, Q9FL01	wall-associated receptor kinase-like 21 precursor
ND_n0000004827	42	GPP00023_1<br>HGP00871_1	no	*Zea mays*	Q5EUC0	thiol oxidoreductase
ND_n0000010444	56	MCP03370_1<br>MAP00510_1<br>GPP00157_1	yes	*Lycopersicon esculentum*	GSHB_LYCES	glutathione synthetase
ND_n0000022177	83	MAP00449_1<br>MIP05371_2	no	*Oryza sativa*	Q40625, Q2QVD7	BZIP transcription factor family
ND_n0000005472	41	GRP00589_1<br>HGP03596_1<br>MHP01915_2	yes	*Arabidopsis thaliana*<br>*Solanum commersonii*<br>*Capsicum chinense*<br>*Brassica juncea*<br>*Cucurbita maxima*<br>*Euphorbia esula*<br>*Oryza sativa*	GST16_ARATH, Q1WW15<br>O22330<br>Q5DUH0<br>Q7XZT0, Q7XZT2, Q7XZT3<br>Q8GT24<br>Q9M533<br>Q56XF1, Q93WM2, GSTH2_ORYSA	glutathione S-transferase
ND_n0000017177	94	MCP03902_1<br>MAP02489_1<br>MPP00736_1<br>MIP02987_1	no	*Arabidopsis thaliana*	Q9ZQ31	hypothetical protein
ND_n0000021399	51	MJP01133_1<br>MHP05238_1	no	*Arabidopsis thaliana*	Q9FGC2	DNA helicase-like
ND_N0000004924	39	MAP00578_2<br>HSP00821_1	no	*Triticum aestivum*	Q84VR8	chimaeric SDH2-RPS14 protein

* species codes are: MI *Meloidogyne incognita*, MA *M. arenaria*, MC *M. chitwoodii*, MJ *M. javanica*, MH *M. hapla*, HS *Heterodera schachtii*, HG *H. glycines*, HC *Haemonchus contortus*, GR *Globodera rostochiensis*, GP *G. pallida*
