# Supplementary material for: On the Extent and Origins of Genic Novelty in the Phylum Nematoda
Source: PLoS Negl Trop Dis. 2008 Jul 2;2(7):e258. doi: 10.1371/journal.pntd.0000258 (PMC2432500; doi:10.1371/journal.pntd.0000258)
Supplement: Alternative Language Abstract S1 — Translation of the abstract into German by Ralf Schmid. Übersetzung der Zusammenfassung ins Deutsche von Ralf Schmid. (0.03 MB DOC) [file pntd.0000258.s008.doc]

**Ausmaß und Ursprung neuer Gene im Phylum Nematoda.**

**James Wasmuth, Ralf Schmid, Ann Hedley & Mark Blaxter**

Im Phylum Nematoda finden sich Spezies mit sehr unterschiedlichen Lebensweisen. So gibt es neben Pflanzen- und Tierparasiten auch freilebende Taxa. Diesen unterschiedlichen Lebensweisen liegen Unterschiede in den exprimierten Genen, einschließlich der Gene die spezifisch mit der Evolution von Parasitismus assoziiert sind, zugrunde. In der vorliegenden Arbeit haben wir EST-Daten für 37 Spezies des Phylums Nematoda analysiert und konnten mehr als 120.000 putative Gene und ihre korrespondierenden Proteine identifizieren. Vereint man diese mit den Proteinsequenzen der vollständig sequenzierten Spezies *Caenorhabditis elegans* und *Caenorhabditis briggsae* erhält man einen Datensatz, der in 65.000 Proteinfamilien, die 40.000 unterschiedliche Proteindomänen enthalten, aufgeschlüsselt werden kann. Wir haben die An- und Abwesenheit dieser Proteinfamilien und Proteindomänen in den verschiedenen Taxa des Phylums Nematoda analysiert und mit Daten, die für andere Phyla vorliegen, verglichen. Mit etwa 500 fehlenden Genen ist speziell in der Linie, die zum Modelorganismus *C. elegans* führt, der Verlust von Genen relativ häufig. Betrachtet man die 56.000 Proteinfamilien und 26.000 Proteindomänen die ausschließlich im Phylum Nematoda vorkommen, so erstaunt die große Menge an „neuen“ Proteinen. Untersucht man dieses Phänomen im Detail, so zeigt sich, daß die Evolution von neuen Proteinfamilien und Proteindomänen im Phylum Nematoda ein Prozess ist der immer noch andauert. Für einige Gene parasitär lebender Spezies gibt es Anzeichen die auf horizontalen Gentransfer vom Wirtsorganismus zum Parasiten hindeuten. Auch findet sich in den parasitär lebenden Spezies ein höherer Anteil an neuen sekretierten Proteinen als in den freilebenden Spezies. Dies deutet darauf hin, daß diese Klasse von Proteinen von grundlegender Bedeutung für die parasitäre Lebensweise im Phylum Nematoda ist. Diese Proteine sind daher ein attraktives Ziel für die Entwicklung von Maßnahmen zur Kontrolle von parasitären Nematoden.
