# Supplementary material for: On the Extent and Origins of Genic Novelty in the Phylum Nematoda
Source: PLoS Negl Trop Dis. 2008 Jul 2;2(7):e258. doi: 10.1371/journal.pntd.0000258 (PMC2432500; doi:10.1371/journal.pntd.0000258)
Supplement: Alternative Language Abstract S2 — Translation of the abstract into French by Douglas Finney. Traduction du résumé en français par Douglas Finney. (0.03 MB DOC) [file pntd.0000258.s009.doc]

**Sur l'étendue et les origines de la nouveauté génique dans le Phylum Nématoda**

**James Wasmuth, Ralf Schmid, Ann Hedley & Mark Blaxter**

*Traduit par Douglas Finney*

Le phylum Nématoda est divers sur le plan biologique comportant aussi bien des parasites de plantes et d'animaux que des taxons non-parasitaires. Le fondement de cette diversité est celle commensurable des gènes spécifiques, y compris les groupes de gènes directement liés à l'évolution du parasitisme. Ici, nous avons analysé les data étendues concernant les 'EST' (disponibles pour 37 espèces de nématodes, la plupart étant des parasites) et nous définissons 120000 gènes putatifs distincts d'où nous avons tiré des traductions robustes de protéines. En combinant ces protéomes a ceux de *Caenorhabditis elegans* et *Caenorhabditis briggsae*, ces protéines ont pu être regroupés en 65,000 familles qui, à leur tour, contiennent 40,000 domaines de protéines distincts. Nous avons tracé l'incidence de domaines et de familles à travers le phylum Nématoda et avons comparé les données relatives aux nématodes à celles disponibles pour d'autres phylums. Une perte de gènes est récurrente; en particulier, nous identifions 5000 gènes qui auraient pu avoir disparu du lignage, ce qui nous mene au modèle *C. elegans*. Nous constatons une prépondérance de nouveauté restreinte aux nematodes, y-compris 56000 familles de protéines ainsi que 26000 domaines. En traçant les plus récentes periodes d'origine de ces nouveaux domaines et familles à travers la phylogénie des nématodes apparait une évolution continue de nouveauté. Un certain nombre d'espèces portait la signature du transfert horizontal de leur organismes d'origine; par ailleurs, les espèces parasitiques contenaient proportionnellement plus de protéines nouvelles, secrétées que celles des espèces non-parasitaires. Il se peut que ces classes de gènes soutiennent des phénotypes parasitiques et ainsi qu'elles pourraient constituer des cibles pour le développement de mesures de contrôle.
